# Atomic structure of a seed-sized gold nanoprism

**DOI:** 10.1038/s41467-022-28829-0

**Published:** 2022-03-09

**Authors:** Yongbo Song, Yingwei Li, Meng Zhou, Hao Li, Tingting Xu, Chuanjun Zhou, Feng Ke, Dayujia Huo, Yan Wan, Jialong Jie, Wen Wu Xu, Manzhou Zhu, Rongchao Jin

**Affiliations:** 1 Department of Chemistry and Centre for Atomic Engineering of Advanced Materials, Anhui University, Hefei, Anhui 230601 China; 2 School of Biomedical Engineering, Research and Engineering Center of Biomedical Materials, Anhui Medical University, Hefei, Anhui 230032 China; 3 Department of Chemistry, Carnegie Mellon University, Pittsburgh, PA 15213 USA; 4 Hefei National Laboratory for Physical Sciences at the Microscale, University of Science and Technology of China, Hefei, Anhui 230026 China; 5 College of Chemistry, Beijing Normal University, Beijing, 100875 China; 6 Department of Physics, School of Physical Science and Technology, Ningbo University, Ningbo, 315211 China

**Keywords:** Structural properties, Nanoparticles, Chemical bonding

## Abstract

The growth of nanoparticles along one or two directions leads to anisotropic nanoparticles, but the nucleation (i.e., the formation of small seeds of specific shape) has long been elusive. Here, we show the total structure of a seed-sized Au_56_ nanoprism, in which the side Au{100} facets are surrounded by bridging thiolates, whereas the top/bottom {111} facets are capped by phosphine ligands at the corners and Br^−^ at the center. The bromide has been proved to be the key to effectively stabilize the Au{111} to fulfill a complete face-centered-cubic core. In femtosecond electron dynamics analysis, the non-evolution of transient absorption spectra of Au_56_ is similar to that of larger-sized gold nanoclusters (*n* > 100), which is ascribed to the completeness of the prismatic Au_56_ core and an effective electron relaxation pathway created by the stronger Au-Au bonds inside. This work provides some insights for the understanding of plasmonic nanoprism formation.

## Abstract (web summary)

The formation pathway of shape-anisotropic nanoparticles is difficult to characterize and not well understood. The authors synthesize a prismatic-shaped Au_56_ nanocluster as possible seed of a prismatic nanoparticle and characterize the structure and ligand bonding motifs, providing insight into the formation and surface protection mechanisms.

## Introduction

In nanoscience research, anisotropic nanoparticles (NPs) are particularly attractive owing to their extraordinary optical, electronic and catalytic properties^1–5^. In 2001, Mirkin and coworkers first reported a bulk solution phase synthesis of Ag nanoprisms and identified quadrupole plasmon resonances^6^. This breakthrough stimulated wide interest in this unique type of anisotropic NPs; for example, subsequent work explored the use of plasmon excitation to control the chemical process of nanoprism growth^7^ and later the plasmon-induced photocatalysis^8–10^, as well as many other scenarios. The structure of nanoprisms (triangular or hexagonal) possesses atomically flat {111} top and bottom facets, whereas the sides are typically {100} or {110} facets^11^. The preferential growth of nanoprisms was explained by the collective effects of the seed structure and plasmon effect^12–14^, the crystal face selective blocking mechanism^15^, the different surface energies of crystal facets and the adsorption of halide ions^16–18^, among the proposed mechanisms for different syntheses. After a long-time debate, it has become clear that halide anions play a significant role in metal nanoprism growth^18,19^. A controlled amount of iodide anions (in addition to Br^–^) leads to the production of Au nanoprisms (tens to hundreds of nm in edge length)^18–20^. Halide anions are also required for the formation of Au and Ag nanorods (tens to hundreds nm in length)^2,18–20^. However, the bonding structure of halides on metal facets is still elusive.

In preparing anisotropic NPs, the early-stage seeds are often critical but remain difficult to study such ultrasmall seeds (typically up to a few nm in size)^2,5,6^. In recent research, atomically precise metal nanoclusters (NCs) of 1–3 nm in diameter (e.g., stabilized by thiolates (SR) or other ligands) have emerged as a new class of nanomaterials, which can serve as models to relate the detailed structures to the assembly and various properties^21–28^, but no relationship between the anisotropic NPs and NCs has been discussed in the literature.

The advantage of pursuing anisotropic NCs with atomic precision lies in the possible determination of total structures (i.e., not only the metal core, but also the surface bonding and arrangement of ligands)^29^, hence, providing atomic-level insights into the “seeds”. Due to the thermodynamics, the vast majority of structurally determined NCs are spherical^30,31^, while anisotropic NCs are rare^32–36^. A rod-shaped Au_8n+4_(SR)_4n+8_ series of NCs is worthy of a comment, as these NCs can be defined as elongated quantum boxes enclosed exclusively by {100} facets (six total), and their growth is by successively adding Au_8_ layers along the [001] direction^37^. Atomically precise gold NCs co-protected by thiolate, phosphine and halides (X = Cl/Br) are more likely of the rod shape^38,39^, and there is no success yet in obtaining the prismatic shape, which is thus highly desirable. The information about the ligand distribution and surface bonding geometry on Au(111) facets of nanoprisms is critically needed but still unknown. This motivated us to obtain atomically precise NCs of prismatic shape in hope of solving their atomic structure, as such information will offer a glimpse of the long sought-after seeds of nanoprisms and also hopefully bridge up the two research domains^1–7,25,40,41^.

Herein, we report the attainment of a prism-shaped [Au_56_(SPh-^*t*^Bu)_24_(P(Ph-4-X)_3_)_6_Br_2_]^2+^ nanocluster of face-centered-cubic (fcc) structure (abbrev. Au_56_ below, and X = CF_3_, Cl, F, or H). This NC possesses ternary ligands that are relevant to plasmonic nanoprism synthesis^6,40^ and also exhibits relatively extended Au(111) facets. By taking advantages of the atomically precise NCs of molecular purity, we successfully crystallized Au_56_ NCs into a macroscopic, coherent superlattice (i.e., a single crystal), and X-ray crystallography analysis determined its total structure. The Au_56_ structure offers a glimpse into the distribution of ternary ligands on the prismatic structure as well as the specific surface bonding. The optical and electronic properties of Au_56_ differ from those of comparable gold NCs^42^ and plasmonic nanoprisms. Overall, the attainment of a prismatic Au_56_ seed sheds light on the formation and surface protection mechanisms of regular sized nanoprisms with plasmon resonances. Future attempts of seeded growth might also offer a possibility for the synthesis of atomically precise, plasmonic nanoprisms.

## Results

### Synthesis and characterization

In a typical synthesis of Au_56_, 0.2 mmol HAuCl_4_·3H_2_O, 0.28 mmol tetraoctylammonium bromide (TOAB), 0.20 mmol phosphine ligand (any of P(Ph-4-CF_3_)_3_, P(Ph-4-Cl)_3_, P(Ph-4-F)_3_, or PPh_3_), and 200 μL tert-butyl-benzenethiol were mixed in solvents of CH_3_CH_2_OH and CH_2_Cl_2_ (v:v = 1:9). Then, 1.5 mmol (CH_3_)_3_CNH_2_·BH_3_, (was added to reduce the gold precursor to NCs, and the reaction was allowed to continue for 12 h. The product was thoroughly washed by n-hexane and further purified by crystallization. The crystallization was conducted by vapor diffusion of n-hexane into a CH_2_Cl_2_ solution of Au_56_ NCs (see Method for details).

**Fig. 1:**
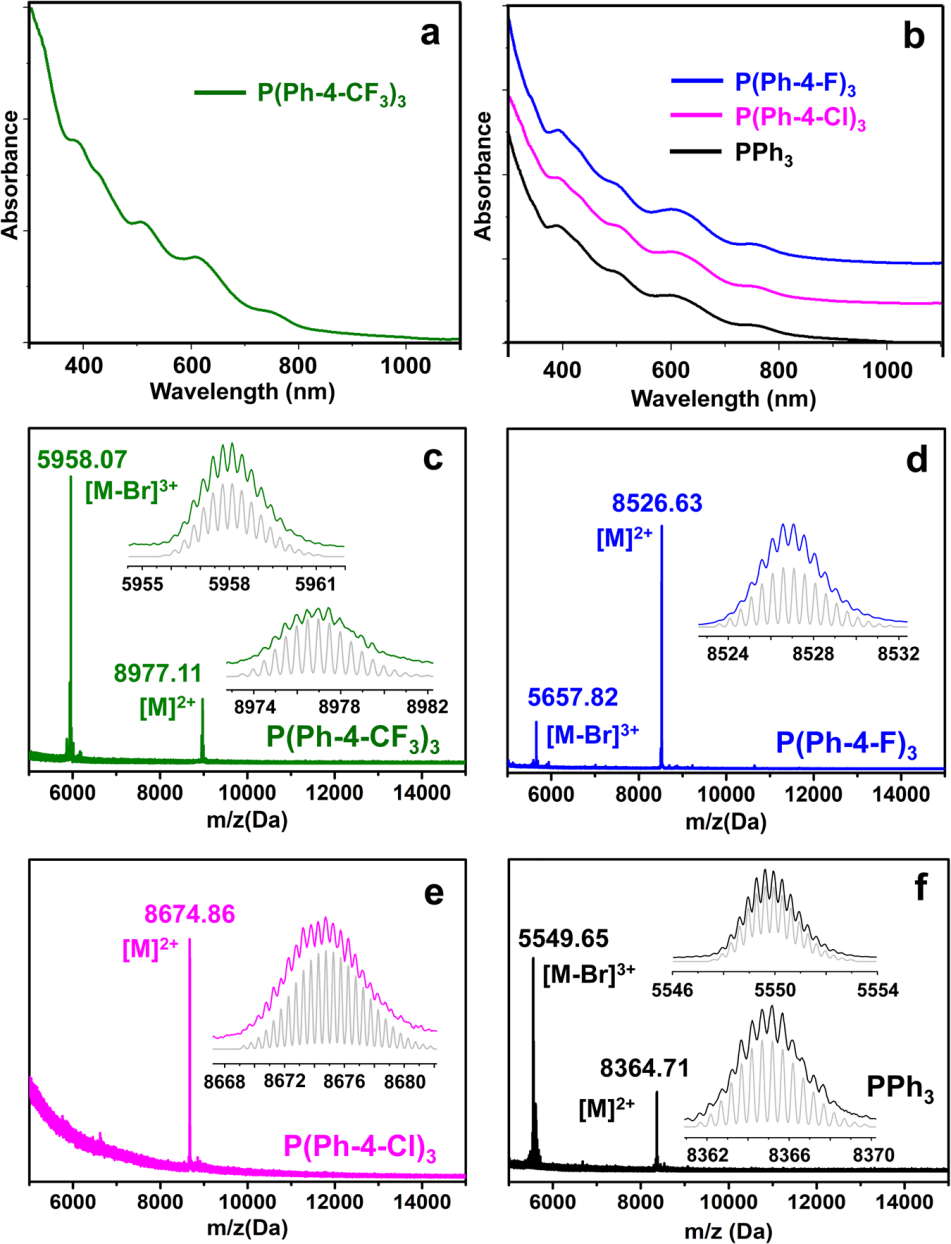
Characterization of Au_56_ NCs containing different phosphines. Experimental UV-vis optical spectra of **a** [Au_56_(SPh-^*t*^Bu)_24_(P(Ph-4-CF_3_)_3_)_6_Br_2_]^2+^ NC and **b** [Au_56_(SPh-^*t*^Bu)_24_(P(Ph-4-X)_3_)_6_Br_2_]^2+^ (X = F, Cl, or H) NCs. **c**–**f** The ESI-MS spectra of the corresponding NCs. The gray profiles in all insets are the simulated isotope patterns which match well with the experimental data of ESI-MS.

The optical absorption spectra of the NCs are slightly different before and after crystallization (Supplementary Fig. 1), indicating the elimination of impurities after crystallization. With redissolved Au_56_ crystals in solution, a distinct step-wise optical absorption spectrum is obtained (Fig. 1a), with peaks at 390, 430, 505, 610, and 750 nm for the case of phosphine = P(Ph-4-CF_3_)_3_. When P(Ph-4-Cl)_3_, P(Ph-4-F)_3_ or PPh_3_ is applied, the spectra of all Au_56_ NCs are the same (Fig. 1b) as the case of P(Ph-4-CF_3_)_3_. The spectra of Au_56_ NCs indicate a nonmetallic state^42^, i.e., the peaks are single-electron transitions (as opposed to the collective electron excitation) and are thus fundamentally different from the in-plane/out-of-plane dipole and quadruple plasmon resonances observed in Au or Ag nanoprisms in metallic state (edge length 40–100 nm, thickness 5–50 nm)^43,44^. The optical absorption onset of Au_56_ is ~1.2 eV (Supplementary Fig. 2). In contrast, differential pulse voltammetry (DPV) gives a HOMO-LUMO gap of 0.93 V (i.e., E_g_ = 0.93 eV); note: the electrochemical gap is 1.21 V (Supplementary Fig. 3, the first oxidation/reduction peak at +0.92 and −0.29 V, respectively) and subtracting the charging energy gives the 0.93 V gap. Thus, Au_56_ NCs have a forbidden HOMO-LUMO transition. On a note, the rod-shaped [Au_25_(SR)_5_(PPh_3_)_10_Cl_2_]^2+^ also had a forbidden HOMO-LUMO transition^45^. Electrospray ionization mass spectrometry analysis (ESI-MS) of the [Au_56_(SPh-^*t*^Bu)_24_(P(Ph-4-CF_3_)_3_)_6_Br_2_]^2+^ indicates the molecular purity (Fig. 1c), so are other Au_56_ NCs with different phosphine ligands (Fig. 1d, e, f).

### X-ray crystallography

**Fig. 2:**
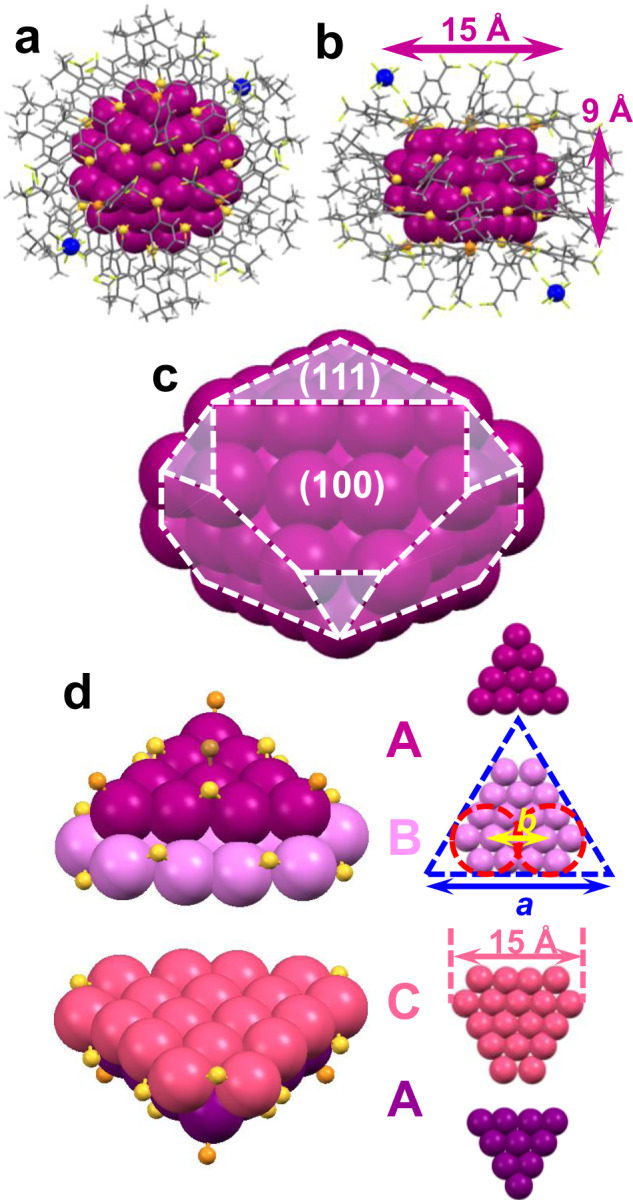
X-ray structure of the Au_56_ NC. **a**, **b** Total structure of [Au_56_(SPh-^*t*^Bu)_24_(P(Ph-4-CF_3_)_3_)_6_Br_2_](SbF_6_)_2_. **c** Prism-shaped Au_56_ and its facets. **d** The four atomic layers in Au_56_. Color code: magenta/violet/coral/purple = Au, yellow = S, orange = P, brown = Br, green = F, blue = Sb, gray = C, white = H.

We further succeeded in crystallization of Au_56_ NCs. We illustrate with the Au_56_ protected by P(Ph-4-CF_3_)_3_, thiolate and Br^−^ ligands. The single crystal data shows that each Au_56_ is associated with two SbF_6_^–^ counterions (Fig. 2a, b, Supplementary Table 1), thus, a 2+ charge of the NC. The 56 Au atoms are arranged into a fcc structure of prismatic shape, hence, an ultrasmall nanoprism (diameter 15 Å × height 9 Å, Fig. 2b). Note that the aspect ratio of nanoprisms is usually above 3 while that of Au_56_ is only ~1.7, indicating that the anisotropic growth of the NC is still at an initial ‘seed’ stage. On the top/bottom of the prismatic Au_56_, 10 Au atoms form a triangular top facet, so is the bottom facet. The side of Au_56_ is comprised of six {100} and six smallest {111} facets (Fig. 2c).

The prismatic Au_56_ structure contains four atomic layers (Fig. 2d, A/B/C/A), i.e., a top Au_10_ triangle, two Au_18_ truncated triangles, and a bottom Au_10_ triangle. The truncated triangular layer shows a degree of truncation, *T* = *b*/*a* = 0.34, consistent with that measured in truncated Ag nanoprisms with edge length ~68 nm^46^, suggesting that the truncation in nanoprisms can be traced to ultrasmall seed-sized NCs. As the A/B bilayer and C/A bilayer are arranged in a staggered manner (*θ* = 60° to each other, Fig. 2d), Au_56_ exhibits a hexagonal-prism shape. If the two halves were arranged in an eclipsed manner, a triangular nanoprism would be obtained with a twin plane (Supplementary Fig. 4)^47,48^.

While the Au_56_ with P(Ph-4-CF_3_)_3_ phosphine ligand had some residual electron densities in the core, which can be attributed to the inadequate absorption correction of gold, which strongly absorbs X-rays. Another two Au_56_ NCs with different phosphine ligands had better crystal quality and were also solved, and all have the same core structure (see the supporting cif files and Supplementary Tables 2 and 3). The counterions to the [Au_56_(SPh-^*t*^Bu)_24_(P(Ph-4-X)_3_)_6_Br_2_]^2+^ (X = F or Cl) are two Cl^–^. It is clear that the ligands and the associated counterions are critical in the orientation control on the assembly of the NCs with the same cluster structure (Supplementary Fig. 5), as the possibly formed hydrogen bonds, the volume of counterions, and their steric and electrostatic effect often cause differences^29^. Specifically, Au_56_ with P(Ph-4-CF_3_)_3_ forms a triclinic unit cell, and Au_56_ with P(Ph-4-F)_3_ gives rise to a monoclinic one, whereas the unit cell of Au_56_ with P(Ph-4-Cl)_3_ is trigonal.

**Fig. 3:**
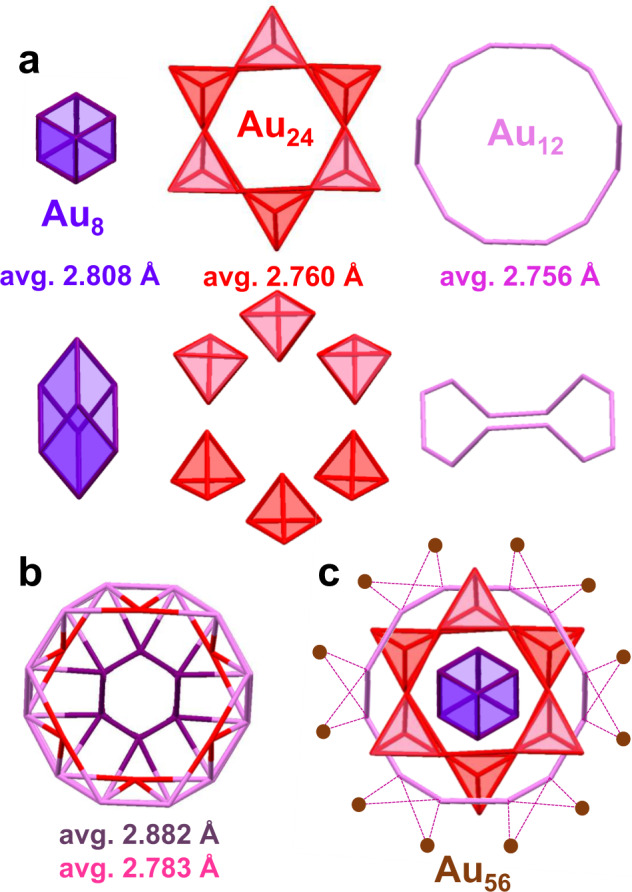
Illustration of the bond lengths in the Au_56_ NC. **a** Top and side views of the rhombohedral Au_8_ kernel (purple), six tetrahedral Au_4_ units (red), and a Au_12_ ring (pink). **b** Connection of the Au_12_ ring with the rhombohedral Au_8_ and six tetrahedral Au_4_ units. **c** Perimetric 12 Au atoms (orange).

According to the Au-Au bond length distribution inside the core, the Au_56_ structure can alternatively be divided into four parts: a central rhombohedral Au_8_ with average bond length of 2.808 Å (Fig. 3a, marked in purple, 2.833(12) Å and 2.784(12) Å, respectively); six Au_4_ tetrahedrons (Fig. 3a, three on the top and another three at the bottom, marked in red) in which the Au-Au bonds are very short (avg. 2.760 Å: 2.763(1), 2.767(10), 2.780(10), 2.764(10), 2.792(10), 2.697(11) Å), and such Au_4_ units were previously observed in other fcc Au NCs^49^; a Au_12_ ring (Fig. 3a, marked in pink, 2.684(11) Å and 2.827(10) Å, respectively) to connect the central Au_8_ with six Au_4_ units surrounding at the waist (Fig. 3b), and the distance between the Au_12_ ring and Au_8_ is 2.873(1) and 2.890(10) Å, respectively, whereas that between Au_12_ and the tip of Au_4_ is 2.722(10) and 2.801(10) Å, respectively; by contrast, the perimetric 12 Au atoms (Fig. 3c, marked in orange) are loosely bonded to the central part of the core. Thus, there is an effective pathway within the Au_56_ NC by having stronger Au-Au bonds (i.e. shorter bond lengths), which presumably endows the NC some interesting dynamics in ultrafast electron relaxation (vide infra).

**Fig. 4:**
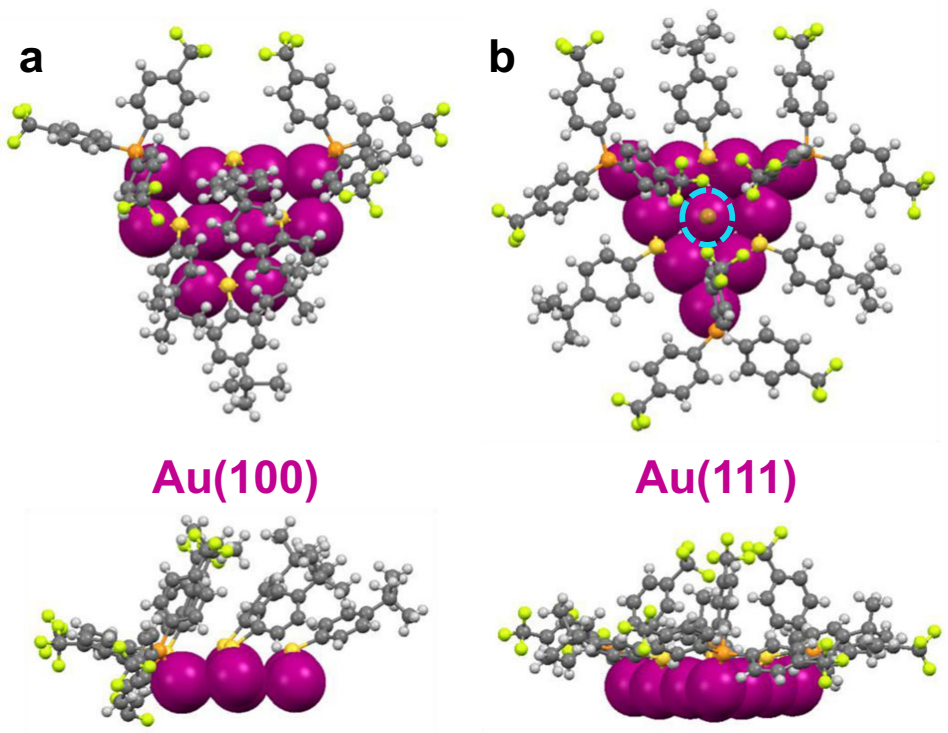
The surface ligand distribution and protection of the Au_56_ NC. Two views of ligands’ assembly on **a** Au(100) facet, and **b** Au(111) facet of Au_56_. Color labels: magenta = Au, brown = Br, orange = P, yellow = S, gray = C, white = H, and green = F.

In previous work on Au or Ag nanoprisms, the surface-protecting ligands and bonding structure were not known. Here, with the Au_56_ structure solved, one can gain a glimpse into such issues. We found that thiolates on Au(100) facets show a bridging mode of bonding, rather than the terminal mode as commonly thought, and the organic parts of the thiolates tilt towards the same direction (Fig. 4a). More importantly, the protection of Au(111) facets by phosphine and bromide is interesting (Fig. 4b). One of the three Ph-4-CF_3_ branches of one phosphine ligand (three phosphine ligands total) points to the central Br^−^ on Au(111), whereas the other two Ph-4-CF_3_ branches stretch outwards (Fig. 4a). The bromide resides on top of the Au atom within Au(111) (Fig. 4a, highlighted by a blue circle) and is critical in stabilizing the Au(111) facets in the Au_56_ NC. It is interesting to see that although I^–^ is critical in directing the formation of gold nanoprisms^18,50^, for Au_56_, Br^–^ is necessary to selectively stabilize the Au(111) facet^51^. We further suppose that, due to the higher surface energy of Au{100} facets, stronger Au-SR bonds are preferred; whereas on the Au{111} facets of lower surface energy, relatively weaker Au-Br bonds are accommodated.

### Electron dynamics

**Fig. 5:**
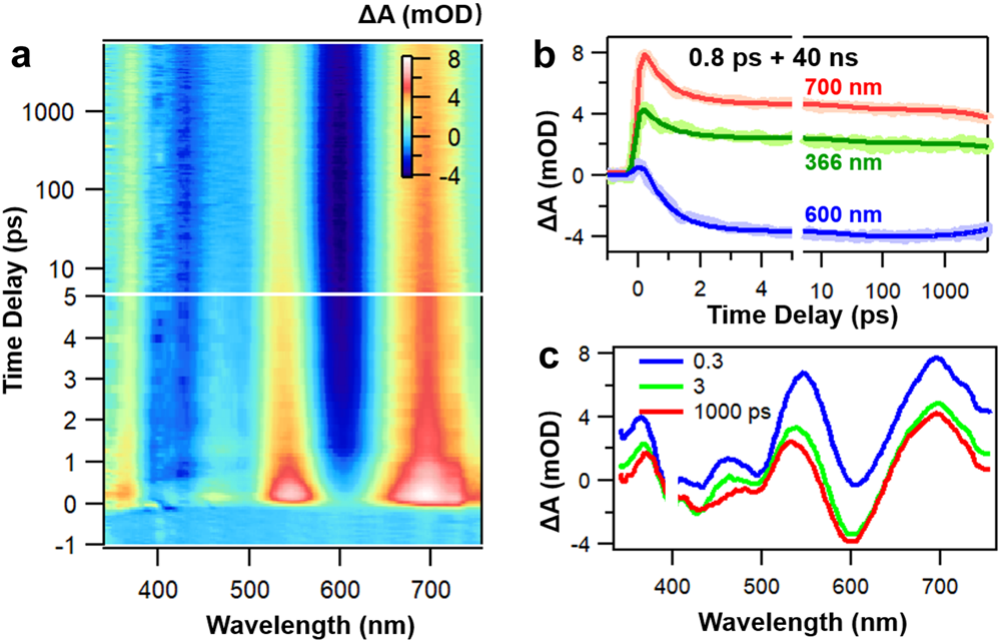
Excited-state dynamics of the Au_56_ NC. **a** TA data map of Au_56_ with 400 nm excitation. **b** TA kinetic traces and corresponding fits at selected probe wavelengths. **c** TA spectra of Au_56_ at selected time-delays.

Due to the completeness of the fcc Au_56_ core structure, we expect distinct electron dynamics in this NC and thus performed femtosecond transient absorption (fs-TA) measurements to probe the excited-state dynamics of Au_56_. According to the TA data map probed between −1 and 7000 ps, after excitation at 400 nm, one can observe positive excited-state absorption (ESA) bands at 365, 540 and 700 nm, together with negative ground-state bleaching (GSB) at 400, 500 and 600 nm (Fig. 5a). Between 0 and 3 ps, a rapid decay for all ESA bands is clear, and the TA spectra of Au_56_ remain almost unchanged between 3 ps and 7000 ps (Fig. 5b, c). Data fitting shows that a fast decay (0.8 ps) followed by a very slow decay (40 ns measured by ns pump-probe, Supplementary Fig. 6) can well fit the TA dynamics at all wavelengths. It is interesting to see that there is almost no spectral evolution in the TA data map; the TA spectral profile at 0.3 ps is almost identical to that at 1000 ps. After normalization, the TA spectra only exhibit a slight blue-shift between 0.3 and 1000 ps (Supplementary Fig. 7). The 40 ns lifetime is comparable to that of small-sized Au NCs (<50 gold atoms)^42^, while the non-evolution of TA spectra is reminiscent of large-sized Au NCs (>100 gold atoms)^42^.

Previous work on similar sized Au_52_(SR)_32_ reported that the profile of ESA bands from higher excited-states is drastically different from that of S_1_ state^52^, which is in contrast to the observation in Au_56_ here. The lack of evolution of the TA spectra of Au_56_ suggests that the ESA of both S_*n*_ and S_1_ states should be a featureless band covering the entire detection range (350–750 nm). Such an observation should arise from the unique packing structure of the Au_56_ NC, i.e., a complete fcc core structure which is larger than that of Au-SR NCs of similar size with motifs, as well as the specific electron motion pathway within Au_56_ in which a Au_12_ ring effectively connects the six Au_4_ units and the central Au_8_ with strong Au–Au bonds. By contrast, in Au_52_(SR)_32_, the Au–Au bond lengths within each Au_4_-coiled helix is shorter than that between the two helices^49^. Besides, the larger fcc core of Au_56_ should eventually lead to more condensed higher excited-states, hence, featureless ESA in the visible range if the negative ground-state bleaching bands were not there.

## Discussion

The bromide on each Au(111) facet should come from TOAB in the reactants, which is similar to cetyltrimethylammonium bromide (CTAB) acting as a cationic surfactant widely used to stabilize colloidal metal NPs^1,2^. A chemical reduction of gold precursor in the presence of Br^–^ is important for synthesizing the Au_56_ prismatic NC with stabilized Au(111) facets, along with the choice of ligands (thiol and phosphine). When we replaced Br^−^ (from TOAB) with Cl^−^ (TBAC: tetrabutylammonium chloride) in the experiment, no Au_56_ NC was formed (Supplementary Fig. 8).

In previous work on the synthesis of regular-sized nanoprisms, phosphine (e.g., bis(*p*-sulfonatophenyl) phenylphosphine dihydrate dipotassium salt) and citrate^6^, as well as thiolate^40^, were used for Ag nanoprisms, whereas CTAB was used to produce Au nanoprisms with the help of iodide^44,53^. The addition of a small amount of I^–^ was previously found to be essential to suppress the growth along the Au[111] direction^18–20^, resulting in nanoprisms of high aspect ratios. We rationalize that for NCs with ~1 nm size, Br^–^ is effective enough to restrain the growth along the Au[111] direction, and the surface bonding of Br^–^ on Au(111) facets is revealed in the structure of Au_56_. Due to the restrained Au[111] growth, the size expansion of Au(111) facet should be favored, however, it is confined by phosphine ligands at the three corners, otherwise the Au{100} facets decorated by thiolates would extend to even larger sizes^32^.

We also noticed that although the atomic packing inside the Au_56_ NC can be considered as fcc, the typical Au-Au bond length of 2.88 Å for bulk Au is not throughout the structure; instead, there are a strongly bonded part and a loosely bonded part. The Au-Au bond lengths within the central rhombohedral Au_8_ and the six tetrahedral Au_4_ units, as well as the Au_12_ ring to connect them together are shorter than the 2.88 Å value in bulk Au. On the other hand, the distances between the Au_4_ units are rather long (3.155 (11) Å), and the perimetric 12 Au atoms are loosely connected to the inner part with avg. 3.047 Å. The effective pathway created by stronger Au-Au bonds inside the Au_56_ NC should be responsible for the special feature of electron relaxation dynamics being similar to what is observed in large-sized Au NCs with more than 100 gold atoms.

In summary, a prism-shaped Au_56_ NC is successfully obtained. The Au(111) facet is protected by three phosphine ligands at the corners and one Br^−^ at the center, indicating the growth mechanism for anisotropic NCs. The presence of Br^–^ is important for stabilizing the Au(111) facets and achieving a complete prism-shaped Au_56_ NC. Such insights are expected to be helpful for understanding the system of conventional NPs. Femtosecond transient absorption measurements of Au_56_ indicates a picture of more congested excited-states than that of similar-sized Au NCs in the literature, which becomes similar to that of those large-sized Au NCs (>100 gold atoms). This can be explained by the strongly bonded pathway inside the Au_56_ with a complete prism in shape. Overall, the atomic structure of seed-sized nanoprism sheds light on the initial stage of nanoprisms and provides useful information for rationalization of the formation and growth mechanism. Future work may explore the use of such precise seeds for possible achievements of atomically precise, larger nanoprisms and thus bridge up the two domains^4–7,40^.

## Methods

### Reagents

Tetrachloroauric(III) acid (HAuCl_4_·3H_2_O, ≥ 99.99% metals basis, Aladdin), tetraoctylammonium bromide (TOAB, 98%, Aladdin), tetrabutylammonium chloride (TBAC, 98%, Aladdin), borane-tert-butylamine complex ((CH_3_)_3_CNH_2_·BH_3_, ≥ 95.0%, Aladdin), tris(4-fluorophenyl)phosphine (P(Ph-4-F)_3_, 98%, Aladdin), tris(4-chlorophenyl)phosphine (P(Ph-4-Cl)_3_, 98%, Aladdin), tris(4-trifluoromethylphenyl)phosphine (P(Ph-4-CF_3_)_3_, 97%, Aladdin), triphenylphosphine, (PPh_3_, ≥ 95%, Aladdin), 4-tert-butylbenzenethiolate (SPh-tBu, ≥ 98.5%, Aladdin), ethanol (HPLC, Aladdin), dichloromethane (HPLC, Aladdin), *n*-hexane (HPLC, Aladdin). All reagents and solvents were commercially available and used as received without further purification.

### Synthesis of Au_56_ NCs

To prepare the Au_56_(SPh-^*t*^Bu)_24_(P(Ph-4-X)_3_)_6_Br_2_ NC, 0.2 mmol HAuCl_4_·3H_2_O, 0.28 mmol TOAB were first dissolved in a mixed solvent of CH_3_CH_2_OH and CH_2_Cl_2_ (v:v = 1:9) for 15 min, and then, 0.20 mmol phosphine (i.e., P(Ph-4-F)_3_, P(Ph-4-Cl)_3_, P(Ph-4-CF_3_)_3_, or PPh_3_) was added. After 30 min, 200 μL HSPh-^*t*^Bu was added, and the solution was stirred for another 30 min. When the solution became almost clear, 1.5 mmol reducing agent ((CH_3_)_3_CNH_2_·BH_3_) was added, and the reaction was continued for 12 h. The product was thoroughly washed by *n*-hexane, and extracted by CH_2_Cl_2_. 20 mg NaSbF_6_ was added to the CH_2_Cl_2_ solution of Au_56_ NCs and mixed for 30 min. The Au_56_ NCs were further purified during the crystallization which was conducted by vapor diffusing *n*-hexane into the CH_2_Cl_2_ solution of the NCs for a week.

### Characterization

Crystals were re-dissolved in CH_2_Cl_2_ and then used as the samples for all the measurements. The UV-vis absorption spectra were collected with an Agilent HP8453 diode array spectrometer. Electrospray ionization (ESI) mass spectra were acquired using a Waters UPLC H-class/Xevo G2-XS QTof mass spectrometer. The sample was dissolved in CH_3_OH/CH_2_Cl_2_ (v:v = 1:1) solution with a concentration of ~0.1 mg ml^−1^. The sample was infused at 20 μL min^−1^ directly. The source temperature was kept at 80 °C with the spray voltage kept at 3.0 kV.

### X-ray crystallographic determination

A suitable crystal was mounted onto a MiTeGen capillary with fluorolube and performed on a STOE STADIVARI diffractometer equipped with CuKα X-ray source (λ = 1.54186 Å). The crystal was kept at 120 K during the data collection. Using Olex2^54^, the structure was solved with the olex2.solve^55^ structure solution program using Charge Flipping and refined with the olex2.refine^56^ refinement package using Gauss-Newton minimization. All the Au, P, Br, S, Cl and Sb atoms were found directly. Remaining non-hydrogen atoms were generated via subsequent difference Fourier syntheses. All the non-hydrogen atoms were refined anisotropically. All the hydrogen atoms were set in geometrically calculated positions and refined isotropically using a riding model. The diffuse electron densities resulted from the residual solvent molecules were removed from the data set using the SQUEEZE routine of PLATON and the refined data was further generated.

### Crystal data

For [Au_56_(SPh-^*t*^Bu)_24_(P(Ph-4-CF_3_)_3_)_6_Br_2_](SbF_6_)_2_ (M = 18425.37 g mol^−1^): triclinic, space group P-1, a = 21.9736(8) Å, b = 24.0131(14) Å, c = 25.8161(12) Å, α = 108.841(4)°, β = 100.243(3)°, γ = 106.585(4)°, V = 11795.6(10) Å^3^, Z = 1, T = 120(2) K, μ(GaKα) = 34.661 mm^−1^, ρ_calc_ = 2.594 g cm^−3^, 69387 reflections measured (7.698° ≤ 2θ ≤ 119.996°), 33856 unique (R_int_ = 0.0630, R_sigma_ = 0.0719), which were used in all calculations. The final R_1_ was 0.0877 (I > 2σ(I)) and wR_2_ was 0.2646 (all data). The residual electron densities left inside the cluster core can be attributed to the inadequate absorption correction of gold which strongly absorbs X-rays.

For [Au_56_(SPh-^*t*^Bu)_24_(P(Ph-4-F)_3_)_6_Br_2_]Cl_2_ (M = 17124.63 g mol^−1^): monoclinic, space group C2/c, a = 45.473 Å, b = 28.445 Å, c = 42.222 Å, α = γ = 90°, β = 121.57°, V = 46531.3 Å^3^, Z = 4, T = 120(2) K, μ(CuKα) = 34.089 mm^−1^, ρ_calc_ = 2.444 g cm^−3^, 216121 reflections measured (7.518° ≤ 2θ ≤ 125°), 37013 unique (R_int_ = 0.0715, R_sigma_ = 0.0348), which were used in all calculations. The final R_1_ was 0.0918 (I > 2σ(I)) and wR_2_ was 0.2735 (all data).

For [Au_56_(SPh-^*t*^Bu)_24_(P(Ph-4-Cl)_3_)_6_Br_2_]Cl_2_ (M = 17346.24 g mol^−1^): trigonal, space group R-3, a = b = 27.1253(17) Å, c = 50.856(6) Å, α = β = 90°, γ = 120°, V = 32406(5) Å^3^, Z = 3, T = 120(2) K, μ(CuKα) = 37.558 mm^−1^, ρ_calc_ = 2.666 g cm^−3^, 30861 reflections measured (8.35° ≤ 2θ ≤ 124.982°), 11310 unique (R_int_ = 0.0518, R_sigma_ = 0.0623), which were used in all calculations. The final R_1_ was 0.0594 (I > 2σ(I)) and wR_2_ was 0.1796 (all data).

### Nanosecond spectral measurement

The nanosecond transient absorption measurements were performed using a nanosecond flash photolysis setup Edinburgh LP920 spectrometer (Edinburgh Instruments Ltd.), combined with a Nd:YAG laser at 355 nm (Surelite II, Continuum Inc.).

### Ultrafast transient absorption

The femtosecond transient absorption spectra were measured at ∼150 fs time-resolution using a commercial femtosecond broadband pump−probe setup (Harpia-TA, Light Conversion)^57^ with an amplified femtosecond Ti:sapphire laser (Coherent Astrella, 7 mJ, 40 fs, 800 nm, 1 kHz). The pumping wavelengths were generated by an optical parametric amplifier (TOPAS-C, Coherent), and the probe pulses were obtained with a CaF2 plate (2 mm thick). The solution samples were in 1 mm optical path quartz cuvettes. Data analysis was performed with R-package TIMP software with the graphical interface Glotaran (Target Analysis model) and CarpetView (Light Conversion).

## Supplementary information

Supplementary Info

## Data Availability

The X-ray crystallographic coordinates for structures reported in this study have been deposited at the Cambridge Crystallographic Data Centre (CCDC), under deposition number CCDC 2015959, 2021449, 2021439. These data can be obtained free of charge from The Cambridge Crystallographic Data Centre via www.ccdc.cam.ac.uk/data_request/cif.
