# Supplementary material for: Atomic structure of a seed-sized gold nanoprism
Source: Nat Commun. 2022 Mar 9;13:1235. doi: 10.1038/s41467-022-28829-0 (PMC8907178; doi:10.1038/s41467-022-28829-0)
Supplement: Supplementary file 1 — Supplementary Info [file 41467_2022_28829_MOESM1_ESM.pdf]

(Supplementary Information)

Atomic structure of a seed-sized gold nanoprism

Yongbo Song[1,4,§*], Yingwei Li[2,§], Meng Zhou[3], Hao Li[1], Tingting Xu[4], Chuanjun Zhou[1], Feng Ke[1], Dayujia Huo[5], Yan Wan[5], Jialong Jie[5], Wen Wu Xu[6], Manzhou Zhu,[1*] & Rongchao Jin[2,*]

[1]Department of Chemistry and Centre for Atomic Engineering of Advanced Materials, Anhui University, Hefei, Anhui 230601, China.

[2]Department of Chemistry, Carnegie Mellon University, Pittsburgh, PA 15213, United States.

[3]Hefei National Laboratory for Physical Sciences at the Microscale, University of Science and Technology of China, Hefei, Anhui 230026, China.

[4]School of Biomedical Engineering, Research and Engineering Center of Biomedical Materials, Anhui Medical University, Hefei, Anhui 230032, China.

[5]College of Chemistry, Beijing Normal University, Beijing 100875, China.

[6]Department of Physics, School of Physical Science and Technology, Ningbo University, Ningbo 315211, China.

[§]These authors contribute equally: Yongbo Song, Yingwei Li.

*email: ybsong860@ahmu.edu.cn; zmz@ahu.edu.cn; rongchao@andrew.cmu.edu

Supplementary Table 1. Crystal data and structure refinement for [Au_{56}(SPh-^{t}Bu)$_{24}$(P(Ph-4-CF_3)$_3$)$_6$$Br_2$]($SbF_6$)$_2$.

Empirical formula	$C_{366}H_{384}Au_{56}Br_2F_{66}P_6S_{24}Sb_2$
Formula weight	18425.37
Temperature/K	120(2)
Crystal system	triclinic
Space group	P-1
a/Å	21.9736(8)
b/Å	24.0131(14)
c/Å	25.8161(12)
α/°	108.841(4)
β/°	100.243(3)
γ/°	106.585(4)
Volume/Å^3	11795.6(10)
Z	1
ρ_{calc}g/cm^3	2.594
μ/mm^{-1}	34.661
F(000)	8249.0
Crystal size/mm^3	0.06 × 0.04 × 0.03
Radiation	CuKα (λ = 1.54186)
2θ range for data collection/°	7.698 to 119.996
Index ranges	$-24 \leq h \leq 24$, $-26 \leq k \leq 20$, $-28 \leq l \leq 28$
Reflections collected	69387
Independent reflections	33856 [R_{int} = 0.0630, R_{sigma} = 0.0719]
Data/restraints/parameters	33856/2431/2266
Goodness-of-fit on F^2	1.013
Final R indexes [I>=2σ (I)]	R_1 = 0.0877, wR_2 = 0.2421
Final R indexes [all data]	R_1 = 0.1121, wR_2 = 0.2646
Largest diff. peak/hole / e Å^{-3}	8.49/-5.75

Supplementary Table 2. Crystal data and structure refinement for [Au_{56}(SPh-^{t}Bu)$_{24}$(P(Ph-4-F)$_3$)$_6$$Br_2$]$Cl_2$.

Empirical formula	$C_{348}H_{384}Au_{56}Br_2Cl_2F_{18}P_6S_{24}$
Formula weight	17124.63
Temperature/K	120(2)
Crystal system	monoclinic
Space group	C2/c
a/Å	45.473
b/Å	28.445
c/Å	42.222
α/°	90
β/°	121.57
γ/°	90
Volume/Å^3	46531.3
Z	4
ρ_{calc}g/cm^3	2.444
μ/mm^{-1}	34.089
F(000)	30544.0
Crystal size/mm^3	0.12 × 0.08 × 0.05
Radiation	CuKα (λ = 1.54186)
2Θ range for data collection/°	7.518 to 125
Index ranges	$-34 \le h \le 52$, $-32 \le k \le 32$, $-48 \le l \le 25$
Reflections collected	216121
Independent reflections	37013 [R_{int} = 0.0715, R_{sigma} = 0.0348]
Data/restraints/parameters	37013/2142/2041
Goodness-of-fit on F^2	1.036
Final R indexes [I>=2σ (I)]	R_1 = 0.0918, wR_2 = 0.2463
Final R indexes [all data]	R_1 = 0.1059, wR_2 = 0.2735
Largest diff. peak/hole / e Å^{-3}	4.52/-5.15

Supplementary Table 3. Crystal data and structure refinement for [Au_{56}(SPh-^{t}Bu)$_{24}$(P(Ph-4-Cl)$_3$)$_6$$Br_2$]$Cl_2$.

Empirical formula	$C_{348}H_{384}Au_{56}Br_2Cl_{20}P_6S_{24}$
Formula weight	17346.24
Temperature/K	120(2)
Crystal system	trigonal
Space group	R-3
a/Å	27.1253(17)
b/Å	27.1253(17)
c/Å	50.856(6)
α/°	90
β/°	90
γ/°	120
Volume/Å^3	32406(5)
Z	3.00006
ρ_{calc}g/cm^3	2.666
μ/mm^{-1}	37.558
F(000)	23178.0
Crystal size/mm^3	0.05 × 0.04 × 0.03
Radiation	CuKα (λ = 1.54186)
2Θ range for data collection/°	8.35 to 124.982
Index ranges	$-31 \leq h \leq 23$, $-29 \leq k \leq 31$, $-57 \leq l \leq 25$
Reflections collected	30861
Independent reflections	11310 [R_{int} = 0.0518, R_{sigma} = 0.0623]
Data/restraints/parameters	11310/732/645
Goodness-of-fit on F^2	1.039
Final R indexes [I>=2σ (I)]	R_1 = 0.0594, wR_2 = 0.1568
Final R indexes [all data]	R_1 = 0.0887, wR_2 = 0.1796
Largest diff. peak/hole / e Å^{-3}	2.73/-3.14

Supplementary figures:

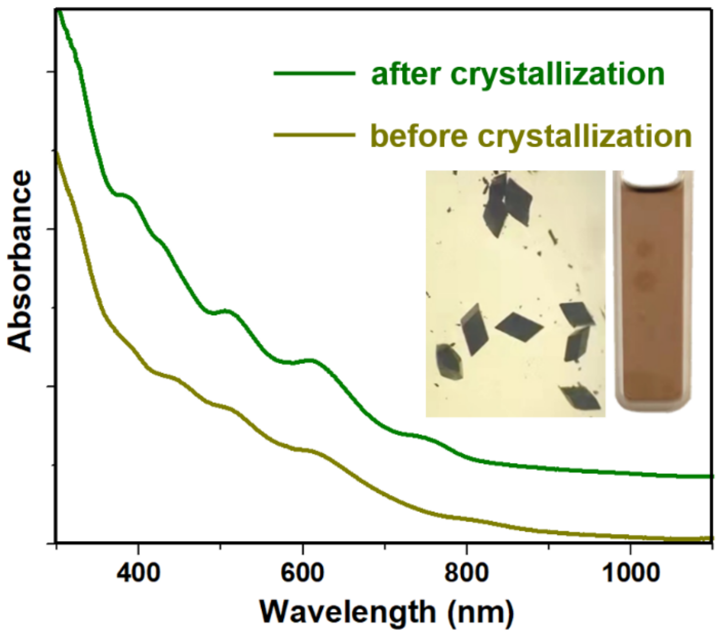

Supplementary Fig. 1 UV-vis optical spectra of $[Au_{56}(SPh\text{-}^{t}Bu)_{24}(P(Ph\text{-}4\text{-}CF_3)_3)_6Br_2]^{2+}$ before and after crystallization. Insets: photographs of single crystals and solution in dichloromethane.

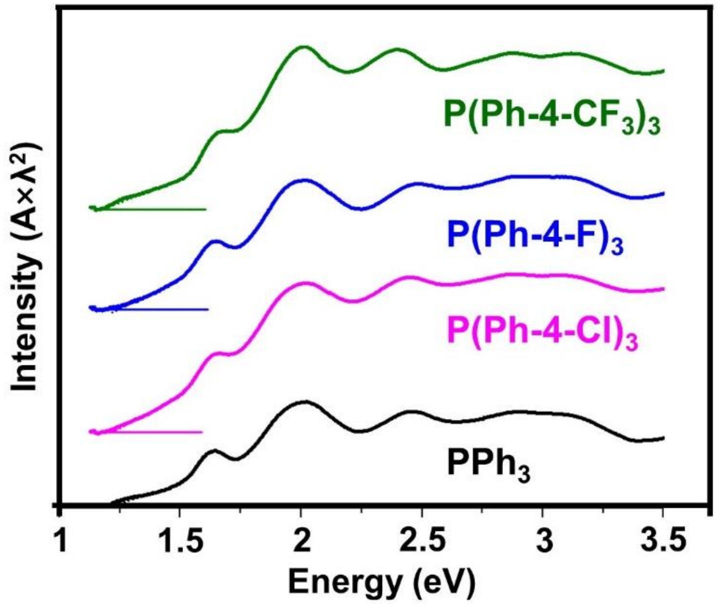

Supplementary Fig. 2 UV-vis optical spectra (on the photon energy scale) of $Au_{56}(SPh\text{-}^{t}Bu)_{24}(P(Ph\text{-}4\text{-}X)_3)_6Br_2$ NCs (green, X = CF_3; blue, X = F; magenta, X = Cl; black, X = H).

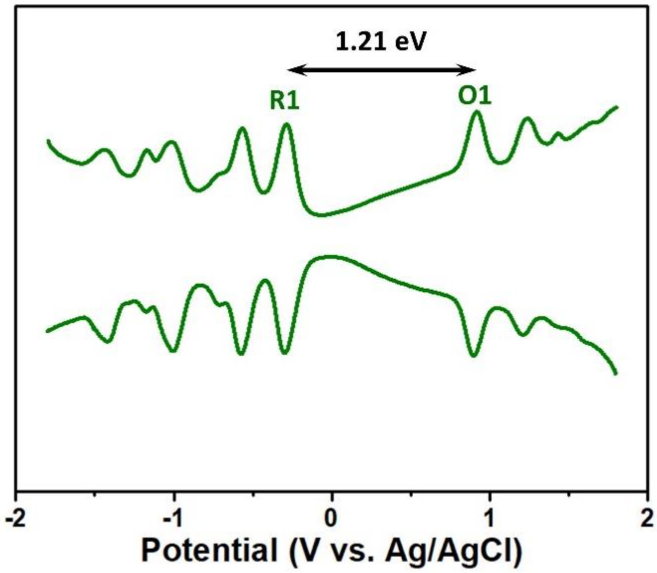

Supplementary Fig. 3 DPV of $Au_{56}(SPh\text{-}^{t}Bu)_{24}(P(Ph\text{-}4\text{-}CF_3)_3)_6Br_2$ in CH_2Cl_2.

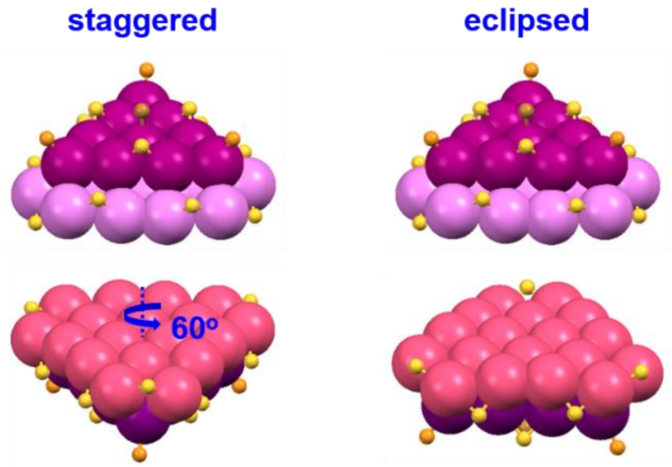

Supplementary Fig. 4 The four atomic layers in Au_{56} in the staggered arrangement (experimental, left) and eclipsed arrangement (hypothetical, right). Color code: magenta/violet/coral/purple = Au, yellow = S, orange = P, brown = Br.

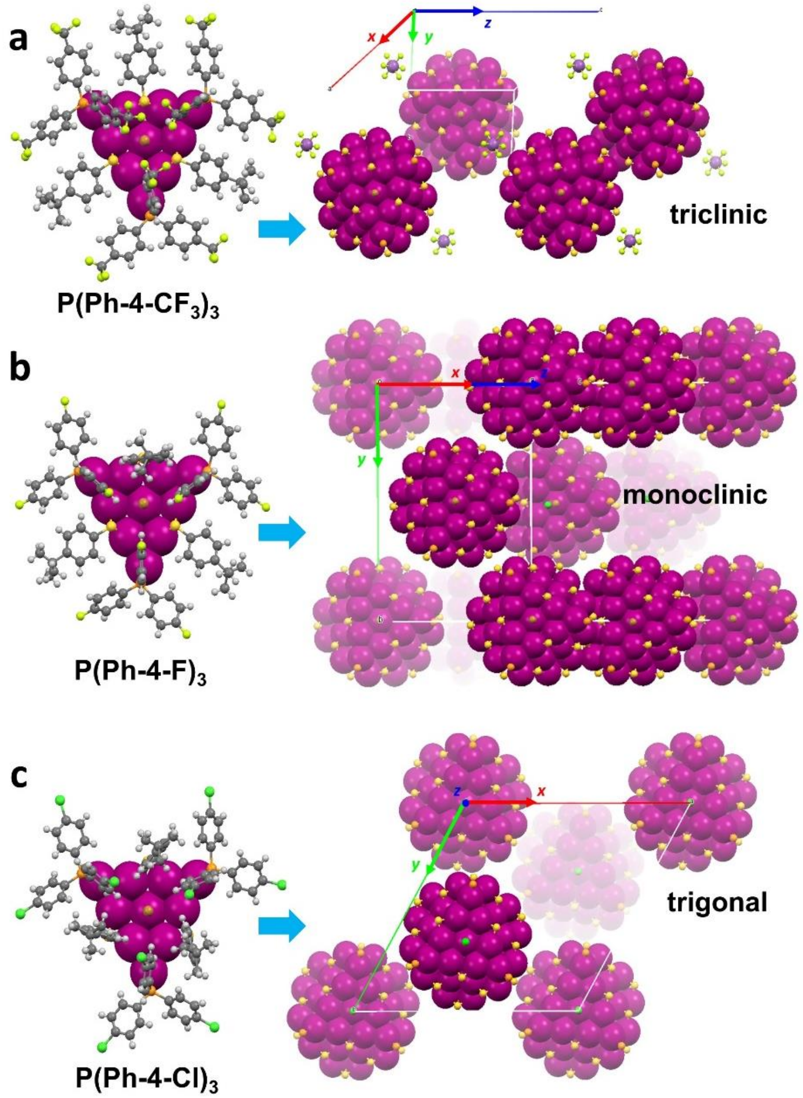

Supplementary Fig. 5 The unit cells of different symmetry formed by Au_{56} NCs with different phosphine ligands: (a) $P(Ph\text{-}4\text{-}CF_3)_3$, (b) $P(Ph\text{-}4\text{-}F)_3$, and (c) $P(Ph\text{-}4\text{-}Cl)_3$. Color code: magenta = Au, yellow = S, orange = P, brown = Br, light green = F, green = Cl, purple = Sb, grey = C, white = H.

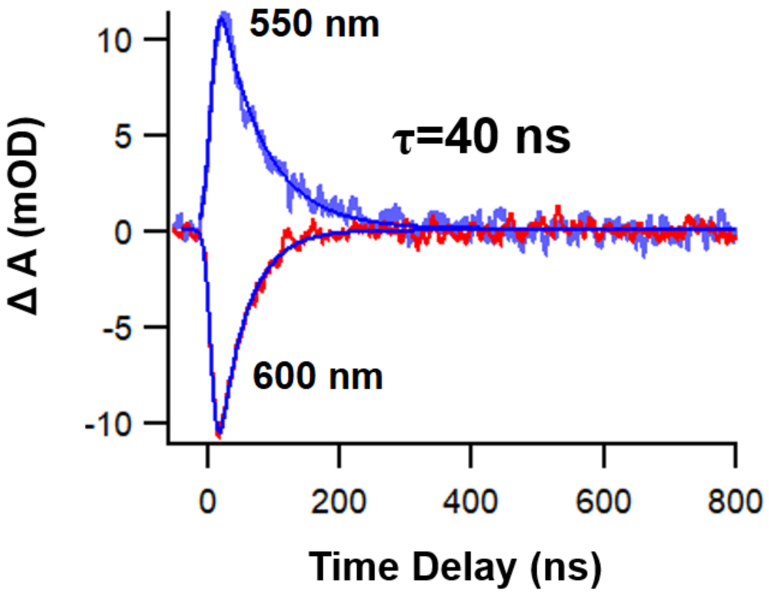

Supplementary Fig. 6 Nanosecond transient absorption decay traces and corresponding fits of Au_{56} measured by flash photolysis.

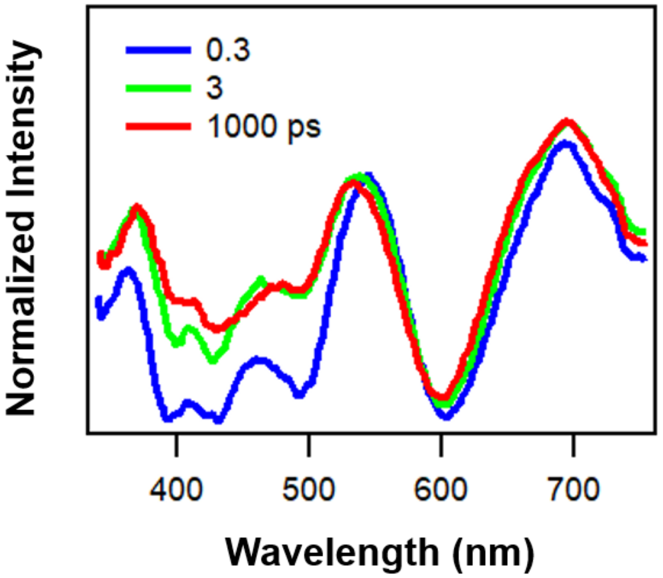

Supplementary Fig. 7 Normalized transient absorption spectra of Au_{56} probed at 0.3, 3 and 1000 ps of time-delay.

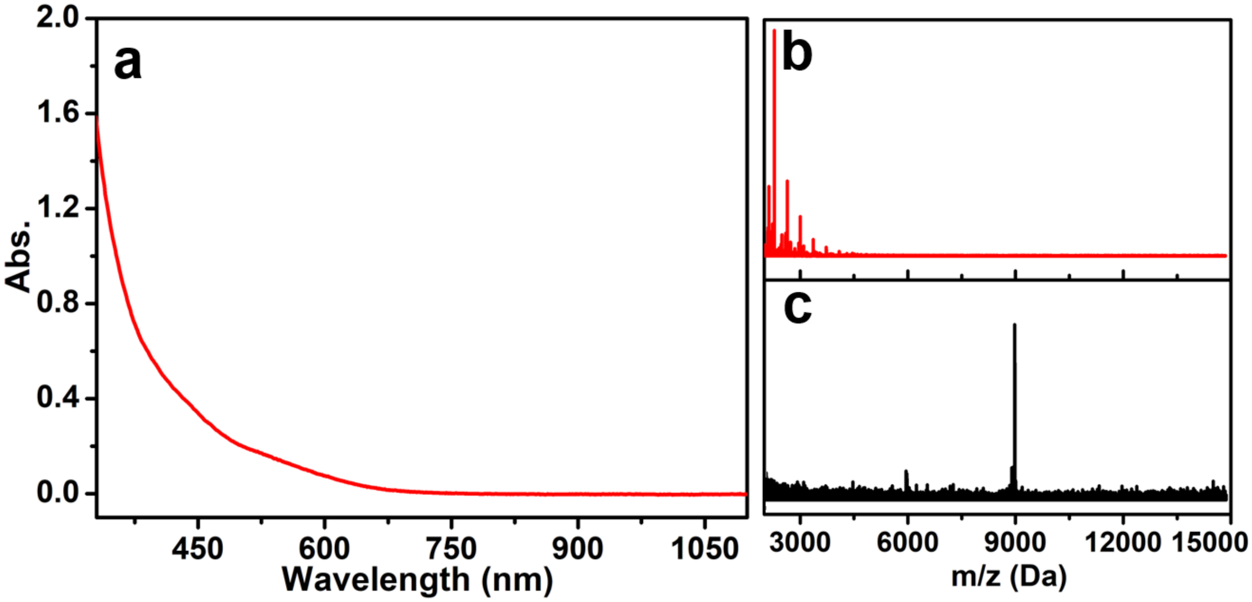

Supplementary Fig. 8 (a) UV-vis and (b) ESI-MS spectra of the as-prepared sample using TBAC; (c) ESI-MS spectrum of the $[Au_{56}(SPh\text{-}{}^{t}Bu)_{24}(P(Ph\text{-}4\text{-}CF_3)_3)_6Br_2]^{2+}$ NCs. Note: ESI-MS spectra were obtained under the same condition.
